# Cytotoxic and genotoxic effects of tert‐butylhydroquinone, butylated hydroxyanisole and propyl gallate as synthetic food antioxidants

**DOI:** 10.1002/fsn3.4373

**Published:** 2024-08-07

**Authors:** Karim Esazadeh, Jafar Ezzati Nazhad Dolatabadi, Hashem Andishmand, Hossein Mohammadzadeh‐Aghdash, Mansour Mahmoudpour, Mohammad Naemi Kermanshahi, Yousef Roosta

**Affiliations:** ^1^ Pharmaceutical Analysis Research Center Tabriz University of Medical Sciences Tabriz Iran; ^2^ Drug Applied Research Center Tabriz University of Medical Sciences Tabriz Iran; ^3^ Research Center for Food Hygiene and Safety, School of Public Health Shahid Sadoughi University of Medical Sciences Yazd Iran; ^4^ Department of Food Hygiene and Safety, School of Public Health Shahid Sadoughi University of Medical Sciences Yazd Iran; ^5^ Miandoab Schools of Medical Sciences Miandoab Iran; ^6^ Food and Beverages Safety Research Center Urmia University of Medical Sciences Urmia Iran; ^7^ Student Research Committee, Nutrition Research Center, School of Nutrition and Food Sciences Tabriz University of Medical Sciences Tabriz Iran; ^8^ Department of Internal Medicne, School of Medicine, Solid Tumor Research Center Imam Khomeini Hospital, Urmia University of Medical Sciences Urmia Iran

**Keywords:** interaction, phenolic food additives, serum albumin, synthetic antioxidants, toxicity

## Abstract

Synthetic food antioxidants such as tert‐butylhydroquinone (TBHQ), butylated hydroxyanisole (BHA), and propyl gallate (PG) have been extensively utilized in different food industries because of their high protectant activities to stop food spoilage and remove foodborne diseases in humans and animals. It would be emphasized that increasing the intake of antioxidants through intracellular may lead to cyto/genotoxicity, and their complex formation with biological molecules eventually accelerate the progress of various diseases like multiple sclerosis, diabetes, neurological disorders, cardiac vascular disease, cancer, etc. Therefore, their toxicity is one of the challenging subjects due to their extensive use in food‐related industries. TBHQ, BHA, and PG antioxidants have cytotoxic, genotoxic, and carcinogenic effects if absorbed in high doses through the gastrointestinal tract. Thermodynamic parameters presented that the hydrophobic bind plays a key role in the complexation of the TBHQ, BHA, and PG with albumin. The molecular modeling results showed that subdomain IIA plays a vital role in the interaction of TBHQ and BHA with albumin. To comprehend the mechanisms of the cyto/genotoxicity effects of these food antioxidants and conformational alterations of albumin macromolecule, we aim to overview numerous types of research that evaluated the cyto/genotoxicity effects of these antioxidants using several procedures.

## Graphical abstract

Synthetic food antioxidant properties have been discussed in detail. Cyto/genotoxicity of synthetic food antioxidants has been reviewed. Interaction of food antioxidants with albumin approved via various methods.

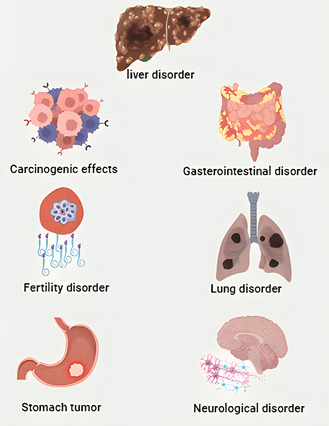

## 1 INTRODUCTION

**FIGURE 1:**
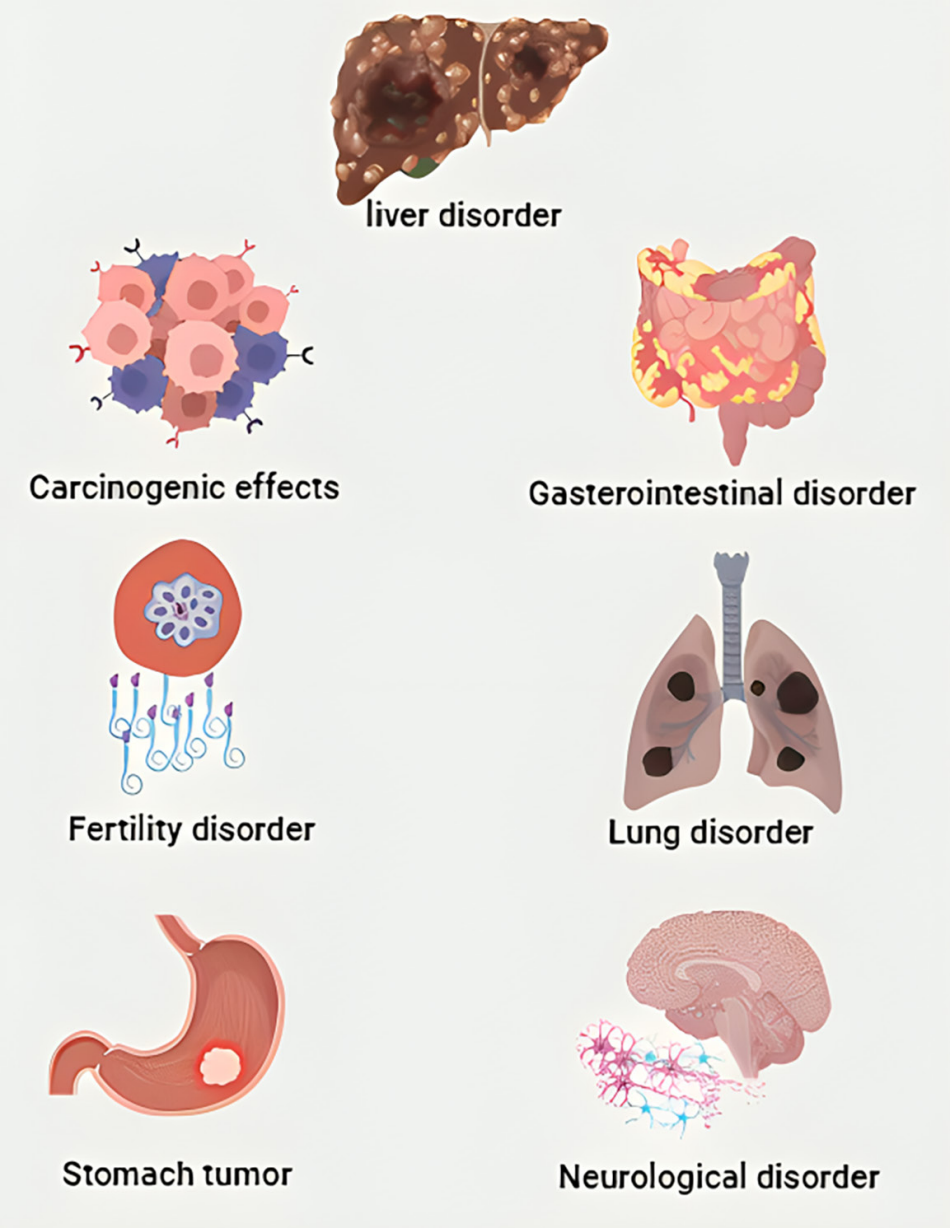
The main disorders caused by long‐term consumption of synthetic antioxidants.

Humans need healthy food to meet the essential requirements of their bodies because foodstuffs and their active components have a substantial impact on the health and nutrition of consumers (Abedini et al., 2023; Fathi et al., 2018; Mohammadzadeh‐Aghdash et al., 2017). Among food pollution, microbiological contaminants are deliberated as the main risks concerning food safety risk assessment and public health. Besides, food deterioration has become an increasingly imperative problem globally, and most food spoilage happens during food storage as a result of chemical reactions or the activity of different organisms (Dehghan et al., 2018). In developed countries, one‐third of the population is affected by foodborne diseases. Several studies have reported that more than 200 physical, chemical, and microbial factors are responsible for the occurrence of these illnesses. The CDC (Centers for Disease Control) in 2011 reported that annually more than 2000 people die because of diseases transmitted via food and around 128,000 people are hospitalized in the USA (Finger et al., 2019). Chemical food additives are largely employed to inhibit food deterioration and remove foodborne diseases in humans and animals (Gallo et al., 2020; Sohrabi et al., 2017). Although chemical food additives are the most important part of the daily diet as functional additives, their use has been restricted by the US (United States), and FDA (Food and Drug Administration). Although some of these chemical antioxidants did not display any harmful effects on the human body after being added to food products, nevertheless, most of them possess a significant impact on both in vitro and in vivo studies. Recent studies showed that a few of these chemical antioxidants are permitted for use in functional foods and prohibited in some countries owing to their harmful effects (Xu et al., 2021). Table 1 displays the properties of synthetic phenolic antioxidants compared to natural ones. Tert‐butylhydroquinone (TBHQ), butylated hydroxyanisole (BHA), and propyl gallate (PG) are increasingly utilized as efficient antioxidants in various industries food, cosmeceutical, and pharmaceutical and their effects on human health should be considered (Shahidi & Zhong, 2010; Xu et al., 2021). Although these components are legally used in food products, they could be detrimental if their body uptake is more than the acceptable levels. TBHQ, BHA, and PG have been broadly employed in preservative processed foods like numerous meat products, unsaturated vegetable oils, edible animal fats, hair products, adhesives, and lubricants to prevent rancidity and spoilage, inhibit microbial growth, and preserve the freshness of the food products (Gulcin, 2020). Cytotoxicity, genotoxicity, and the interaction of food antioxidants with albumins like serum albumin are of attention for investigators to comprehend its possible danger to human health (Xu et al., 2021). Figure 1 shows the main disorders caused by long‐term consumption of synthetic antioxidants. In this context, it is shown that cyto‐genotoxicity of TBHQ, BHA, and PG in various cell lines using different procedures investigated in recent years and their interaction with serum albumin can cause conformational alterations in biological samples. Likewise, the stimulation of intracellular and extracellular accelerators, like oxidative stress, and amyloid fibril creation of serum albumin upon interaction with antioxidants caused various illnesses like diabetes, obesity, and gastric cancers. Herein, in this study, we will outline cytotoxicity, genotoxicity, and complex creation of aforementioned food antioxidants with serum albumin. Cytotoxicity and albumin interactions of these dietary antioxidants have been commonly evaluated by numerous researchers in the past decades. Due to the prevalent usage of synthetic food antioxidants, it is important to analyze the cyto‐genotoxicity results of food antioxidants and interaction of them with albumin using numerous techniques to comprehend the mechanisms of their toxicity and interaction (Xu et al., 2021).

**TABLE 1:** Synthetic and natural phenolic antioxidants: A comparison of their properties.

Synthetic phenolic antioxidant	Natural phenolic antioxidant	References
Widely usage (on a large industrial scale)	Restricted usage (in the research phase)	Liu and Mabury (2020) and Zeb (2020)
Antioxidant capacity ranges from moderate to high	Diverse and extensive antioxidant effects	Santos‐Sánchez et al. (2017) and Xu et al. (2021)
Cost‐effective	Costly	Zeb (2020)
The quality of these items remains unaffected by cooking heat	The quality of these items remains affected by cooking heat	Martelli and Giacomini (2018) and Xu et al. (2021)
Don't offer a variety of products	Offer a variety of products	Berdahl et al. (2010) and Zeb (2020)
High safety concern	Consider a safe material	Andishmand et al. (2023) and Martelli and Giacomini (2018)
Do not exhibit antioxidant effects on human tissues	Exhibit antioxidant effects on human tissues	Liu and Mabury (2020)
They can be used as coloring or flavoring agents	They only prevent spoilage	Martelli and Giacomini (2018) and Zeb (2020)
This can potentially result in negative impacts on the human body	Own multiple health benefits	Liu and Mabury (2020)
Limited solubility in water	Diverse solubility profiles	Augustyniak et al. (2010)
Prohibition of the use of certain ones	Growing adoption and broadening the scope of applications	Augustyniak et al. (2010)
Some of them are not metabolized and stored in fat tissue	Metabolized	Rodrigues et al. (2020) and Xu et al. (2021)

## 2 PROPERTIES OF SYNTHETIC PHENOLIC ANTIOXIDANTS

### 2.1 TBHQ

#### 2.1.1 Sources

TBHQ is a synthetic phenolic compound with chemical formula: C10‐H14‐O2, CAS number 1948‐33‐0, and E number E319 which metabolically be constructed from 3‐tertbutyl‐4‐hydroxyanisole. It is known as tert‐butylhydroquinone or tertiary butylhydroquinone and can be produced through many methods but mainly it can be chemically synthesized. It is the hydroquinone derivative, replaced with a tert‐butyl element. TBHQ is insoluble in water and soluble in ethanol. It's a light‐colored crystalline product with a slight odor. Also, TBHQ protects foods that contain iron from discoloration (Van Esch, 1986).

#### 2.1.2 Benefits

TBHQ is the most effective antioxidant in different foodstuffs including various meat products, edible animal fats, unsaturated oils, and oils versus oxidative deterioration. Thus, delaying the expansion of rancidity and extending of shelf life of these products at a level of less than 0.02 percent is possible using this additive. TBHQ neither change the color of food products even in the iron existence nor modify the taste and smell of the food substrates to which it is added. It is also used as a stabilizer to inhibit auto‐polymerization in some foodstuffs. The utilization of this substance extends to industrial applications where it serves as an antioxidant, commonly found in various cosmetic items such as blushers, eye shadows, and lipstick, typically at a concentration below 0.1%. TBHQ can be found in an excessive variety of foods containing lipids. It is an approved food antioxidant in the United States, the European Union, Australia, and many other regions. A major benefit of various foods is their stability at high temperatures, and the maintenance of antioxidant capacity during the process and after heating and frying at 180°C or above (Beker et al., 2016; Hojjati‐Najafabadi et al., 2020).

#### 2.1.3 Side effects

It has some side effects in large amounts like changes in DNA structure and stomach tumors in experimental animals (Blundell et al., 2022). Nowadays, the exerted nephrotoxicity influence of the TBHQ in rats and cortical apoptosis and traumatic brain damage in mice has been proven by several authors. Current research demonstrates that TBHQ could affect the 8‐hydroxydeoxyguanosine production in calf thymus DNA as an outcome of the generation of ROS (reactive oxygen species). Even though recent studies have observed that TBHQ triggers cancer cell death at high doses, they have also paradoxically found that TBHQ increases carcinogenic effects when studied in laboratory animals. However, the TBHQ effect on breast cancer has not been entirely explored. It has been pointed out that there is probably a relationship between the occurrence of various diseases like breast cancer and the extensive application of TBHQ in processed foods (Kashanian & Dolatabadi, 2009).

#### 2.1.4 Limitations

TBHQ is the most commonly used food antioxidant owing to its low cost, high performance, and wide availability. It has previously been assessed via the former SCF (Scientific Committee on Food), but there was still a need for further information. The acceptable daily intake (ADI) of TBHQ ranges from 0 to 0.7 mg per kg of body weight. If a mixture of TBHQ, PG, and BHA is used, the separate levels should be decreased proportionately. Table 2 shows the acceptable daily intake and the results of different cellular and molecular methods to investigate the effects of TBHQ, BHA, and PG (Eskandani et al., 2014).

**TABLE 2:** Acceptable daily intake and results of different cellular and molecular methods to investigate the effects of TBHQ, BHA, and PG.

Food additives	ADI mg/kg	Cell viability	Apoptotic cells	Necrotic cells	Albumin binding site	References
TBHQ	0 and 0.7	28%	34%	38%	Subdomain IIA	Eskandani et al. (2014) and Shahabadi et al. (2011)
BHA	0 and 0.5	7.51%	87.40%	5.09%	Subdomain IIA	Gu et al. (2021) and Vandghanooni et al. (2013)
PG	0 and 0.5	20.66%	76%	3.34%	Subdomain IB	Dolatabadi et al. (2014) and Hamishehkar et al. (2014)

### 2.2 BHA

#### 2.2.1 Sources

BHA is also named 4‐methoxyphenol with chemical formula: C11‐H16‐O2, CAS number 25013‐16‐5, and E number 320. It is composed of two isomeric organic compounds, 3‐tert‐butyl‐4‐hydroxyanisole, and 2‐tert‐butyl‐4‐hydroxyanisole that could be chemically created through an interaction of methoxyphenol with isobutene (Additives et al., 2018).

#### 2.2.2 Benefits

BHA has been frequently utilized as a food antioxidant to stabilize and protect the nutritive rate and other organoleptic properties such as animal feed harvests and freshness/flavor of foodstuffs for many years. It is applied to decrease free radical formation and prohibit food deterioration and lipid oxidation, especially in fried foods. BHA is utilized as a preservative in food for human consumption, animal feeds, veterinary medications, and related food items. Moreover, it is commonly perceived as a safe component when present in a quantity below 0.02% in food products with oil or fat constituents. BHA is normally approved as a safe (GRAS) by the FDA once utilized by good manufacturing practice. In addition, the toxicity and radiation poisoning of numerous xenobiotics and mutagens can be prevented using BHA antioxidants (Adeyemi, 2021; Dawidowicz et al., 2015).

#### 2.2.3 Side effects

Some literature displayed that BHA reveals an extensive range of biological activities in in vivo models (Xu et al., 2021). According to the literature, BHA can act as a tumor motivator or a tumor promoter in different animal tissues (Imbabi et al., 2021). In this regard, hydroxyanisole‐triggered carcinoma development in the stomach of laboratory animals like hamsters, mice, and rats once fed constantly in large amounts, and the proliferative impact of BHA in mammals and pigs' esophagus has been reported. Vandghanooni et al. (2013) reported that BHA can stimulate cytotoxicity and another study demonstrated that BHA in smooth muscle cells induces HO‐1 gene expression. Acute toxicity of BHA, immune response, stimulation of phase II detoxifying enzymes, and particularly tumor‐promoting activities have been proved by recent studies (Branen, 1975). Therefore, it should be noted that the widespread use of BHA in foods can lead to cytogenetic and molecular toxicity.

#### 2.2.4 Limitations

About 50 states allow the BHA application as a potential antioxidant for food consumption at acceptable levels. BHA is quickly absorbed from the gastrointestinal tract and its metabolites are excreted in the urine and feces of animal species. According to the FDA, ADI for hydroxyanisole is between 0 and 0.5 mg/kg body weight (b/w) per day (Additives et al., 2018; Emerton & Choi, 2008).

### 2.3 PG

#### 2.3.1 Sources

PG is also called propyl 3,4,5‐trihydroxybenzoate with chemical formula: C10‐H12‐O5, CAS number 121‐79‐9, and E number 310. It is generated by extraction with propane‐1‐ol from pods of the Tara tree and subsequent purification. PG also is a chemical that can be produced by a reaction of propyl alcohol with gallic acid accompanied by condensation to eliminate excess alcohol (Nguyen et al., 2021).

#### 2.3.2 Benefits

PG is broadly used as a potential synthetic antioxidant in numerous industries such as cosmetics and food products to inhibit fat rancidity and oxidative deterioration, particularly to protect polyunsaturated fats (Dolatabadi & Kashanian, 2010). PG is commonly utilized together with other food antioxidants, e.g., BHA and TBHQ. In contrast to PG, gallates exhibit a prolonged chain structure, providing enhanced solubility in fats and increased stability during food processing. A study revealed that PG antioxidants can affect lipid peroxidation in sunflower and sorghum seedlings. Gallate serves as a crucial antioxidant‐based hepatoprotector in both in vitro and in vivo models (Javaheri‐Ghezeldizaj et al., 2023). The use of PG might be a hopeful option to control pericarp frying, inhibit tyrosinase activity, and increase the shelf life of commercially harvested fruits (Xu et al., 2021). It has been reported that preventing nucleic acid synthesis and the respiration system inhibits the growth mechanism of microorganisms by PG, and recently the cytoprotective and antioxidative properties of PG have been studied (Dolatabadi et al., 2014).

#### 2.3.3 Side effects

Due to the prevalent use of PG antioxidants in various industries, it has extensively been studied in both in vitro and in vivo models to recognize cytotoxicity and genotoxicity properties (Silva et al., 2017). Considering the low PG toxicity, it has undesirable effects on the regular functions of target cells and tissues. According to recent studies, PG can contribute to mitochondrial dysfunction and subsequent cellular respiration inhibition, which might cause ATP (adenosine triphosphate) depletion (Dolatabadi et al., 2014; Shahidi & Ambigaipalan, 2015). The toxicological behaviors of chemical antioxidants like PG were reported using DNA, DAPI, and MTT fragmentation tests. While PG is recognized as an antioxidant substance, recent research has shown that it cannot only reduce the level of intracellular glutathione but also increase the volume of ROS by inhibiting various enzyme activities including superoxide dismutase (SOD) and catalase. PG material, when present with Cu (II), causes single‐strand breaks with minimal DNA damage due to metal ions. However, when PG transforms into GA (gallic acid) in the existence of metal ions, it is known to induce DNA breaks. It has been emphasized that GA plays a critical role in the cytotoxic and carcinogenic effects of gallate (Additives et al., 2020; Javaheri‐Ghezeldizaj et al., 2023).

#### 2.3.4 Limitations

The use of PG is permitted in all types of oils, fats, and fat‐containing foods with extreme limits of gallate such as BHA and TBHQ. PG is listed as a GRAS substance by the US federal FDA with a total concentration of 0.02% of the fat or oil content of food products. Therefore, if mixtures of TBHQ, BHA, BHT, gallates, and PG are utilized, the individual limits should be reduced proportionally (Emerton & Choi, 2008).

## 3 CYTO/GENOTOXICITY STUDIES OF SYNTHETIC PHENOLIC ANTIOXIDANTS

The side effects of synthetic food additives on human health have been reported in recent studies (Xu et al., 2021). Various research results showed that consuming high amounts of synthetic food additives can lead to neurological and gastrointestinal disorders (Zeb, 2020). In several studies, it has also been observed that synthetic food additives change the composition of the intestinal microbiota and accelerate gastrointestinal disorders (Xu et al., 2021). It is supposed that the consumption of synthetic additives such as hydroquinone, hydroxyanisole, and gallate significantly reduces the cell layer and has a role in chronic inflammatory diseases such as colitis and chronic disease, colon cancer, obesity, and diabetes (Wang et al., 2021). In this regard, a meaningful assessment of the genotoxicity of food additives can only be done after considering any confounding cytotoxicity effects. Food additives were shown to be cytotoxic when administered orally to laboratory animals (Additives et al., 2020). Several procedures are employed for viable cells measurement via 3‐(4, 5‐Dimethylthiazol‐2‐yl)‐2, 5‐diphenyltetrazolium bromide (MTT), trypan blue, dual dye viability, histopathology, acridine orange, Evans blue staining, and neutral diffusion techniques (Ramasamy & Pakshirajan, 2022). In recent years, the capacity of living cells to absorb certain dyes to remove dead cells has been used as a cytotoxicity indicator. However, the MTT test is currently applied to determine the cytotoxicity of various food additives (Andishmand et al., 2024).

Genotoxicity defines the feature of chemical substances that damage the genetic information inside cells and cause mutations in the genetic material that may induce cancer (Menz et al., 2023). Synthetic food additives are materials that directly or indirectly contribute to human foodstuffs. For this reason, studies on the genotoxicity effects of food additives along with their effects on human health are perfectly abundant (Xu et al., 2021). Genotoxicity effects of these additives have been reported exactly in different cell lines and cause a structural change in the subcellular. There are different methods for in vitro genotoxicity assessment of food additives including alkaline comet, DNA (deoxyribonucleic acid) fragmentation, and DAPI (4′,6‐diamidino‐2‐phenylindole) staining techniques. Study results showed that these additives have increased the frequency of DNA damage and chromatin fragmentation in treated cells in comparison to negative control cells (Pinter et al., 2020).

### 3.1 Cyto/genotoxicity of TBHQ

**FIGURE 2:**
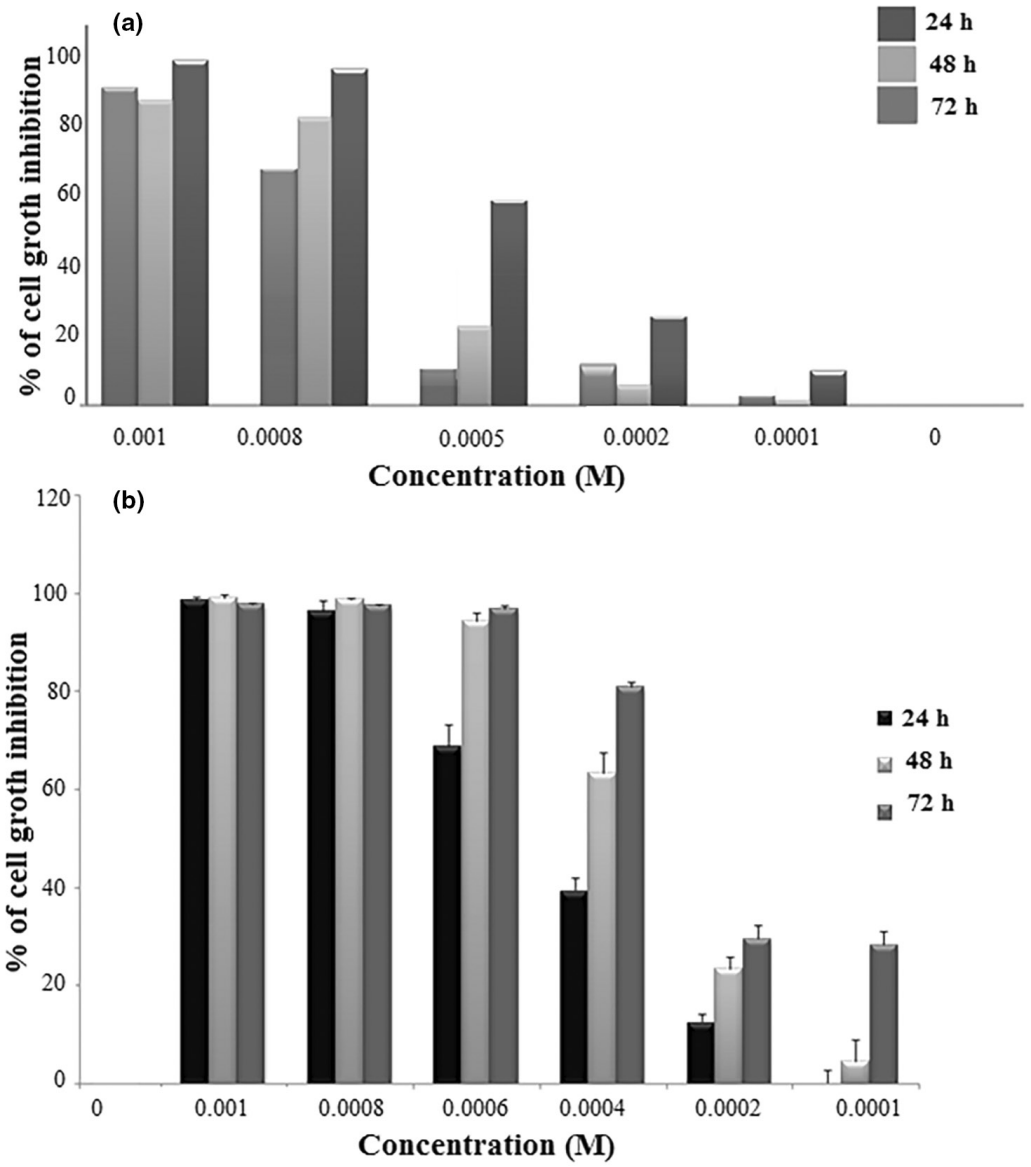
Cytotoxicity effects of TBHQ (a) and BHA (b) on the A549 cells (reprinted with permission [28,37]).

**FIGURE 3:**
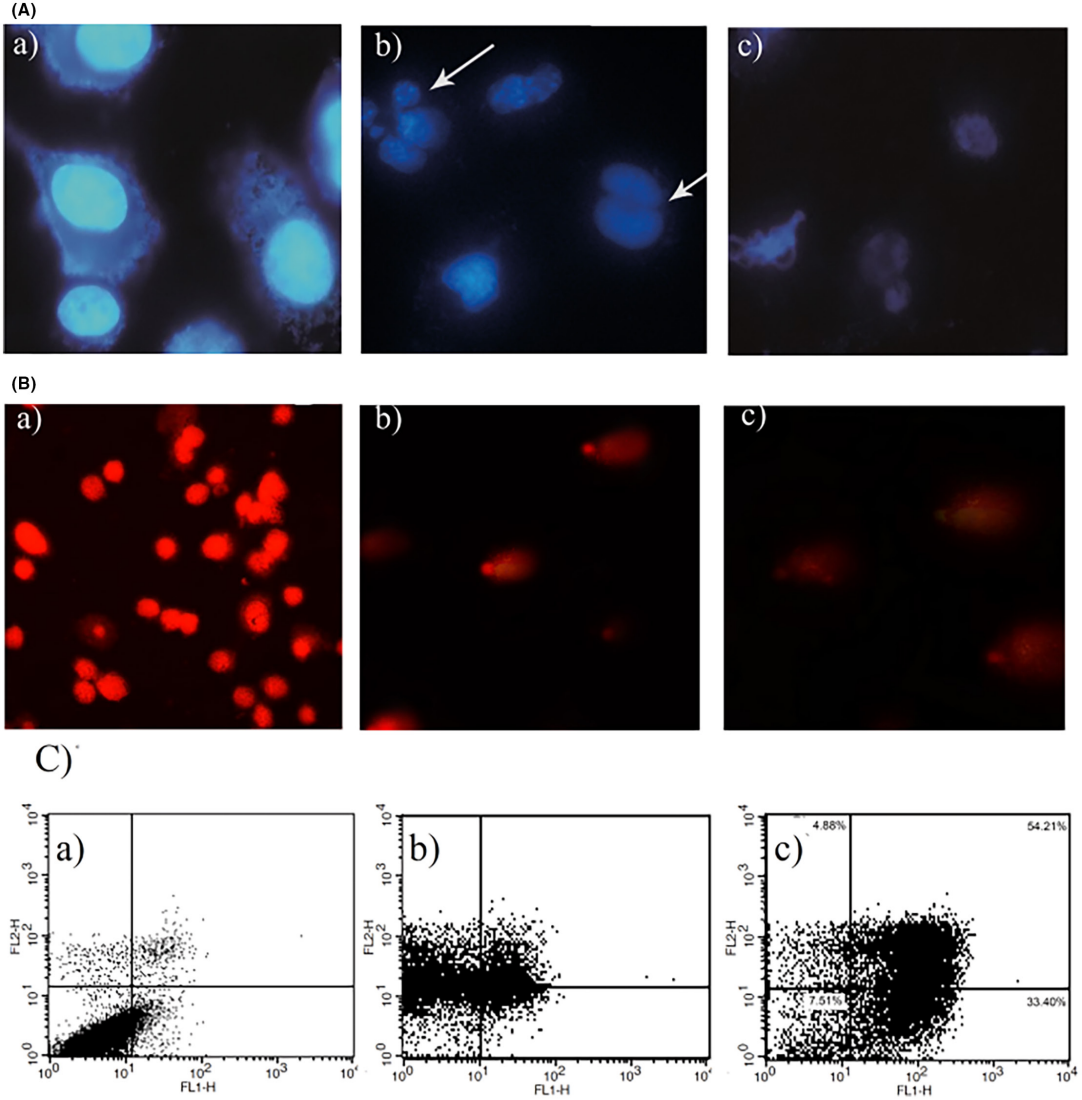
(A) FITC‐labeled annexin V flow cytometric result of negative control (a), TBHQ (b), and BHA (c) on the A549 cell line. (B) Fluorescent microscopy results of negative control (a), TBHQ (b), and BHA (c) on the A549 cell line stained with DAPI. (C) Photographic illustration results of negative control (a), TBHQ (b), and BHA (c) on the A549 cell line (reprinted with permission (Eskandani et al., 2014; Vandghanooni et al., 2013).

The cytotoxicity of TBHQ additives on A549 and HUVEC cells was assessed through the MTT test, and genotoxicity effects were assessed using DAPI staining, DNA fragmentation, and alkaline comet procedures in vitro. The outcomes of the above test exhibited that TBHQ was capable of triggering cytotoxicity in both case‐study cells in a dose‐ and time‐dependent manner as presented in Figure 2. Flow cytometry results confirmed incidence of early/late apoptosis in the treated cells with TBHQ (Figure 3a). Also, the morphology of breaked DNA and DAPI‐stained cells based on electrophoresis exhibited distinct fragmentation in the DNA rings and chromatin of cell's treated with TBHQ (Figure 3b). Indeed, the mechanism of TBHQ toxicity was understood using alkaline comet and DNA fragmentation methods (Figure 3c) (Eskandani et al., 2014). Another study demonstrated that TBHQ could induce cytotoxic and genotoxic effect in HUVECs. Braeuning and coworkers reported that incubation of murine 3T3 cells with TBHQ (300 μg/mL) induces the death of treated cells (Braeuning et al., 2012). Previous studies displayed that stromal cells from bone marrow are sensitive to toxicity caused by benzene metabolites like TBHQ and also it could trigger QR (quinone reductase) in the bone marrow of stromal cells as a consequence of stromal toxicity (Twerdok et al., 1992). In addition, the TBBQ (tert butyl benzoquinone) as a major oxidation product of TBHQ, in the time‐ and dose‐dependent manner declined the cell's activity and suppressed DNA synthesis by modulating the S/G2 cell cycle transition (Ye et al., 2018). The cell proliferation, cell cycle progression, apoptosis, and modification of biological activities are cyto‐genotoxic effects of TBHQ at high concentrations (Sanidad et al., 2016). Among all the hydroquinones tested, TBHQ was the most toxic, followed by 2,5‐di(tert‐butyl)‐1,4‐benzohydroquinone and hydroquinone in rat hepatocytes, and this toxicity can arise from the oxygen consumption rate through each hydroquinone (Liu & Mabury, 2020). Another study suggested that TBHQ metabolites such as TBBQ decreased the viability of RAW 264.7 cells and induced autophagy in a dose‐dependent manner (Meng et al., 2020).

### 3.2 Cyto/genotoxicity of BHA

Among numerous food additives, BHA is widely employed for inhibiting rancidity or oxidation of food products. Due to its extensive application in the field of food products, all individuals who partake in them effectively consume boxed food items. Previously, cytotoxicity effects of BHA were tested using flow cytometry and MTT methods whereas genotoxicity was estimated by DAPI staining, alkaline comet, and DNA fragmentation techniques in A549 cells in vitro, and it had been shown that BHA decreased the A549 cells growth in a time‐ and dose‐dependent way as presented in Figure 3b. Early and late apoptosis in the incubated cells with BHA was specified based on the flow cytometry assay. The morphology of DAPI‐stained cells exhibited considerable alteration in the DNA and chromatin inside the cell's nucleus, which was presented in Figure 3b. In addition, DNA fragmentation has been observed using the alkaline comet method (Figure 3c) (Vandghanooni et al., 2013). In this regard, the mechanism of BHA toxicity was inferred by alkaline comet assays and DNA breakage due to significant intracellular DNA fragmentation. Additionally, relevant studies displayed that oral consumption of BHA induces cytotoxicity and genotoxicity, increases the progress of neoplastic lesions and preneoplastic in the bladder and stomach of mice, causing forestomach squamous cell carcinomas in lab animals, and boosted tumor promotion at high concentrations (Yu et al., 2000). Yu et al. (2000) showed that stimulating mitochondrial permeability alteration upon binding of the BHA with cell mitochondria could lead to cytochrome‐c releasing, apoptosis, and necrosis occurrence. The direct contact of BHA with macromolecules such as DNA and reactive intermediates production was reported in in vitro models (Xu et al., 2021). A new work proposes that BHA can induce cytotoxic and genotoxic effect in root tip cells of *A. cepa*. Indeed, it should not be consumed in packed food products in higher dosages and even low dosages may be detrimental due to the persistent use of these antioxidants. Besides, its accumulation in the body may cause cytotoxic, genotoxic, and carcinogenic effects on humans (Pandey & Kumar, 2021). In addition, enhancement of cell proliferation, stimulation of oxidative DNA damage, and cyto/genotoxicity effects of BHA on human lymphocytes were investigated in vitro as well (Xu et al., 2021).

### 3.3 Cyto/genotoxicity of propyl gallate

One of the main important properties of synthetic phenolic antioxidants is their possible toxicity effects in in vivo and in vitro models (Xu et al., 2021). Imbalance in the cell's signaling and physiology is the foremost cause of cytotoxicity and apoptosis of PG compound. The toxicity of PG is one of the challengeable matters deriving from its extensive use in various food industries. The cytotoxic effects of PG were investigated on A549 lung cancer cells and showed that PG decreased the A549 cell growth in a time‐ and dose‐dependent manner. Besides, early and late apoptosis in incubated cells with PG was confirmed by flow cytometry test. The morphologic changes, chromatin breaks, and DNA fragmentation were detected by the DAPI staining producers (Hamishehkar et al., 2014). Upon former studies, the cyto/genotoxicity of PG using the alkaline comet test was observed. It has been noted that cell respiration disorder arising from mitochondrial dysfunction occurs due to a reduction in operating system (OS)‐related enzyme action including catalase and superoxide dismutase (SOD) owing to increase in food antioxidants concentration such as PG (Han & Park, 2009). In addition, Nakagawa et al. (1995) revealed that PG and gallates are toxic to hepatocytes and its cyto/genotoxicity impacts might be owing to mitochondrial impairment. The carcinogenesis and mutagenesis properties of PG have been described to be both enhancing and suppressing effects (Rosin & Stich, 1980). Ultimately, Tayam and Nakagawa (2001) reported that the induction of SCEs (sister‐chromatid exchanges) and CAs (chromosomal aberrations) are cyto/genotoxicity properties of gallate in CHO‐K1 cells.

## 4 BASIC EXPLANATION ABOUT PHENOLIC FOOD ADDITIVES‐DNA INTERACTION

Food additives are widely employed for a variety of reasons, such as preservation, pigmentation, and enhancing sweetness. These compounds are incorporated to prevent or postpone the degradation of nutrients due to bacterial, enzymatic, or chemical alterations in edibles, as well as to prolong the duration of storage and the overall excellence of food products. Synthetic food antioxidants occupy a significant place in the food industry (Nanditha & Prabhasankar, 2008; Thorat et al., 2013). Recently, additives have fascinated the attention as probable causes of numerous diseases such as hepatic and nephritic failures, mutagenic and cancer, etc. There are various studies that displayed the cytotoxicity and genotoxicity of diverse additives in various cell lines. Among the numerous food additives, TBHQ, BHA, and PG exhibit the capacity to establish molecular complexes with the DNA (nucleic acid) framework (Pandir, 2016; Yang et al., 2023; Yilmaz et al., 2014).

### 4.1 TBHQ–DNA interaction

Several authors have reported that the interaction of TBHQ with DNA likely occurs through a mechanism of intercalation, exhibiting a high affinity that could potentially lead to damage in CT‐DNA (Wang et al., 2020). The characterization of TBHQ's interaction with DNA through hyperchromism in the absorption spectrum was conducted. The observed typical binding mode implies potential damage to the DNA helix structure following the intercalation of TBHQ. A fluorescence spectral analysis was carried out to investigate the interaction between TBHQ and DNA, revealing a decrease in DNA emission intensity with increasing TBHQ concentration, providing evidence of their interaction (Dolatabadi & Kashanian, 2010; Wang et al., 2020). Kashanian et al.'s study in 2009 demonstrated the ability of TBHQ to intercalate between base pairs of the DNA molecule, as evidenced by hyperchromism in the UV absorption band of DNA, an increase in the specific viscosity of DNA, and a reduction in TBHQ fluorescence with increasing concentrations of DNA. The electrochemical method has recently been widely employed as a straightforward and efficient approach to investigate the interaction of food compounds with the DNA molecule (Dolatabadi & Kashanian, 2010). Nagai et al. (1996) displayed that the interaction of TBHQ with DNA can be a cause for the creation of 8‐hydrox deoxyguanosine in calf thymus DNA owing to its binding with TBHQ. Another study employed multispectroscopic technique, viscosity measurements, chemometrics, molecular docking, and gel electrophoresis assays to examine the interaction of TBHQ with cDNA, leading to the alteration of DNA conformation from B‐form to A‐form. The study above examined the potential of TBHQ and Cu (II) in causing DNA damage through the application of gel electrophoresis technique. It was found that the interaction between TBHQ and DNA is largely driven by hydrophobic forces with moderate affinity. A noteworthy biomarker for DNA damage resulting from oxidative stress, 8‐hydroxy‐2′‐Deoxyguanosine (8‐OHdG), was generated at 37, 60°C, and pH 7.4. This process involved the reaction of TBHQ with 2′‐deoxyguanosine‐5′‐monophosphate within DNA, and the analysis was carried out using reversed‐phase HPLC assay with a UV detector set at 254 nm wavelength (Handayani et al., 2017).

### 4.2 BHA–DNA interaction

In recent researches, the interaction of BHA with calf thymus DNA at physiological condition was studied by spectrofluorometric, voltammetric, viscosimetric assays, and other techniques. It has also been discovered that BHA has the potential to intercalate with the base pairs of the DNA molecule (Dolatabadi & Kashanian, 2010; Kashanian & Ezzati Nazhad Dolatabadi, 2009). A fluorescence spectrum study of the interaction between BHA and DNA was performed, and the emission intensity of DNA was reduced by increasing the amount of added BHA impurities, indicating that the BHA‐DNA interaction exists (Figure 3b). Fluorescence studies have also been used to determine the binding constant between BHA and DNA, as reported by Kashanian and Ezzati Nazhad Dolatabadi in 2009. In this regard, the value of binding constant (K_b_) was high sufficient to show that BHA can intercalate in within DNA molecule. Recently thermodynamic parameters of BHA and DNA complex at four different temperatures 283, 293, 303, and 310 K through Van't Hoff equation were determined (Kashanian & Dolatabadi, 2009). The ∆*H* and ∆*S* values of the BHA–DNA formation were −89.02 kJ/mol and −211.21 J/mol, respectively. As it is clear the response is exothermic and enthalpy preferred, the negative entropy values approve the intercalative constant mode of BHA and DNA (Wang et al., 2014). It has been reported that BHA interaction with DNA is through intercalation mode using fluorimetry method. Stern–Volmer equation displayed a Ksv (fluorescence quenching by DNA) rise with reducing pH reaching a minimum at pH 3. This proposes that interaction between BHA and DNA at low pH is an electrostatic interaction mode. Molecular docking studies by other studies showed that BHA bound more sensitively to the G‐C‐rich regions of ctDNA but analysis of CD spectra showed that BHA‐DNA binding changed the B‐like DNA conformation to A‐like one. The binding constants for BHA and DNA formation were 2.03 × 10^4^, 1.92 × 10^4^, and 1.59 × 10^4^ L/mol at 298, 304, and 310 K, respectively (Wang et al., 2014; Zhang et al., 2023).

### 4.3 PG–DNA interaction

PG is another imperative antioxidant that is a natural molecule. Its ability to intercalate a DNA molecule has not been observed. By increasing the amount of added DNA, the emission intensity of the PG molecule decreases, indicating that there is an important interaction between PG and DNA (Javaheri‐Ghezeldizaj et al., 2023). For PG‐DNA interaction, the K_b_ value was not high enough to indicate that PG can be intercalated between DNA base pairs. The K_b_ values clearly emphasize the remarkably low affinity of PG for DNA (Table 1). In fact, PG binds to CT‐DNA through lateral binding, such as electrostatic binding. PG can also cause conformational changes in CT‐DNA, but not as severe as other food additives. Thermodynamic parameters of PG‐DNA formation at four different temperatures (283, 293, 303, and 310 K) were determined (Javaheri‐Ghezeldizaj et al., 2023; Nguyen et al., 2021). DH and DS values of PG‐DNA complex were 67.91 kJ/mol and +227.58 J/mol, respectively (Table 1). The positive entropy confirms the non‐intercalated binding mode of PG to DNA. The specific viscosity of the DNA sample clearly decreases with the addition of PG. In fact, the interaction of PG with DNA causes the contraction of the DNA helix, the shortening of the DNA molecule, and the reduction of DNA viscosity. Therefore, it can be confirmed that the interaction between PG and DNA occurs through external binding and non‐intercalation (Javaheri‐Ghezeldizaj et al., 2023).

## 5 INTERACTION OF SYNTHETIC ANTIOXIDANTS WITH SERUM ALBUMIN

Synthetic phenolic antioxidants have been commonly evaluated for their toxicological and biological properties. Thus, once the toxicity data of these phenolic antioxidants becomes available, their use in different industries ought to be reviewed (Mohammadzadeh‐Aghdash et al., 2017). Interactions between synthetic chemicals and biological macromolecules have received increasing attention from several researchers in recent years. Among the various macromolecules, serum albumin is the most abundant soluble protein in the mammalian circulatory system, which has several important physiological functions (Fathi et al., 2016; Sohrabi et al., 2017). Therefore, reviewing the interaction between synthetic chemicals and albumins like human serum albumin (HSA) is significant for many researchers to comprehend its possible hazard to public health.

### 5.1 TBHQ interaction with serum albumin

**FIGURE 4:**
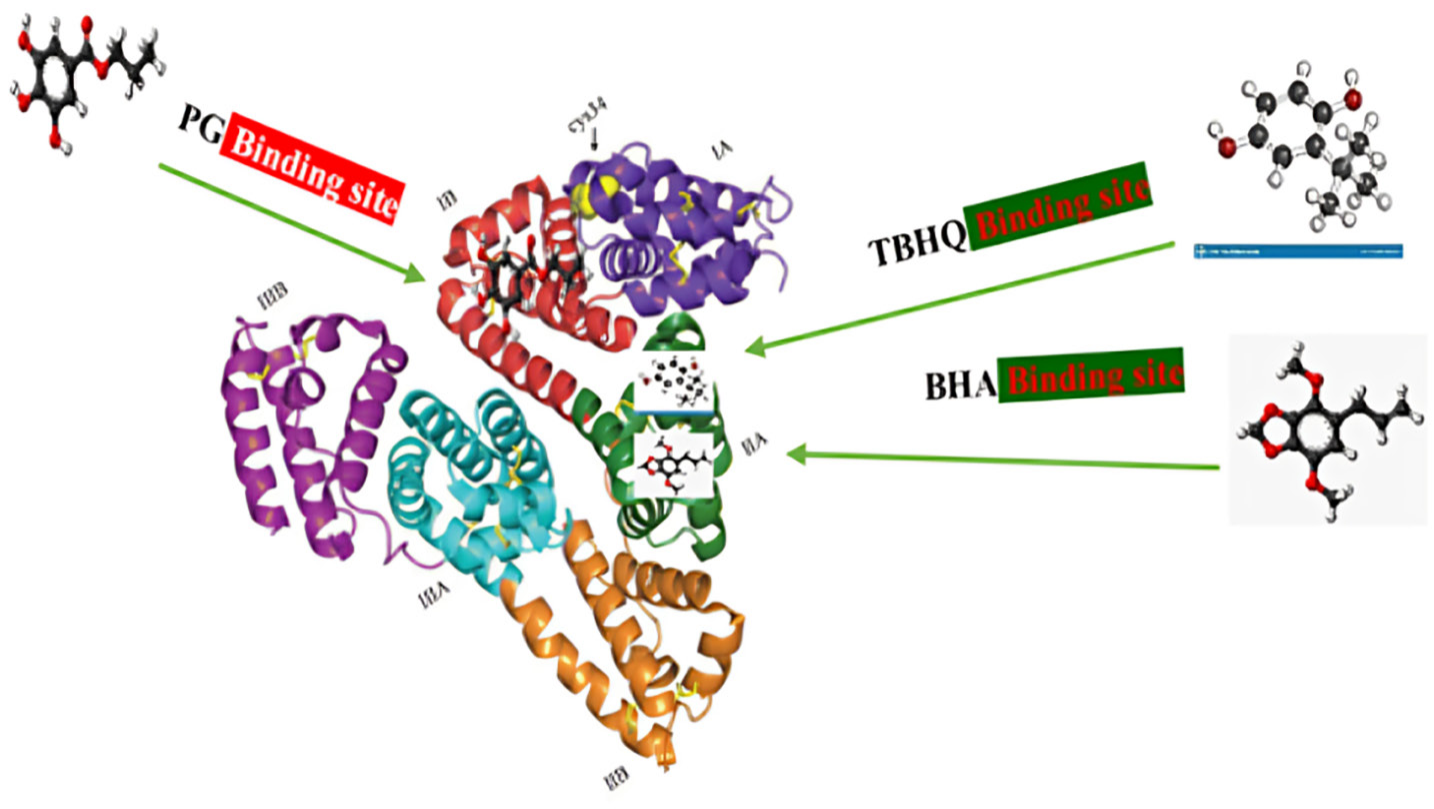
Interaction results of serum albumin complex with TBHQ, BHA, and PG. The aforementioned antioxidants and albumin binding sites are shown by an arrow.

The binding of TBHQ with serum albumin was considered by various procedures such as spectrophotometry, spectrofluorimetry, CD (circular dichroism), FT‐IR (Fourier‐transform infrared spectroscopy), and SPR (surface plasmon resonance) under physiological conditions (Zhang et al., 2012). Previous studies' results proved that the quenching procedure of albumin with TBHQ was a static mode, and the attained results of the SPR technique showed that TBHQ can dynamically interact with albumin with high affinity which has been led to the dose–response sensogram attained for TBHQ. Numerous binding parameters including a positive value of ∆*S* and a negative value of ∆*H* specified that hydrogen bond and hydrophobic forces have main roles in the TBHQ and albumin binding (Fathi et al., 2016; Shahabadi et al., 2011). The binding sites on HSA are located in IIIA and IIA, which were studied by Zhang et al. (2008). Therefore, it is proposed that TBHQ mainly interacts with a hydrophobic site placed in subdomain IIA of albumin (Bi et al., 2005). Figure 4 illustrates the interaction results of TBHQ additives and albumin macromolecule as shown by an arrow.

### 5.2 BHA interaction with serum albumin

After absorption by the gastrointestinal tract, BHA can enter the blood through non‐covalent bonds and interact with serum albumin, which affects its free concentration, metabolism, distribution, and absorption. On the other hand, the BHA binding with albumin triggers the structural alterations of albumin, influencing the biological activities of it (Shahabadi et al., 2011; Sohrabi et al., 2017; Sun et al., 2017). The BHA binding with albumin and effects of its on human health were evaluated by several authors using various procedures such as molecular docking, multi‐spectroscopy, and CD techniques. A previous study disclosed that the mechanism of albumin fluorescence quenching by BHA is in static mode. The binding constant and thermodynamic parameters of recent studies showed that BHA and albumin interact with each other in a relatively stable conformation via hydrophobic procedure and molecular docking experiments proved that site I within subdomain IIA plays a key role in the interaction of BHA and albumin macromolecule as presented in Figure 4 (Al‐Shabib et al., 2017; Gu et al., 2021; Wu et al., 2015).

### 5.3 PG interaction with serum albumin

PG is an example of a phenolic antioxidant agent, which has prevalent use in the food and pharmaceutical industries. Dolatabadi et al. investigated the PG interaction with albumin under physiological conditions. The obtained results showed that PG could meaningfully bind to albumin with high affinity, by which the intrinsic fluorescence of albumin is strongly quenched and the thermodynamic variable showed that the hydrophobic binding plays a key role in the complexation of the PG with albumin and molecular modeling results indicated that site IB play major role in the binding of PG with albumin (Dolatabadi et al., 2014; Fathi et al., 2016; Javaheri‐Ghezeldizaj et al., 2020). As demonstrated, the maximum hydrophobic forces on PG interaction with albumin happen in the subdomain IB owing to the existence of hydrophobic amino acids including L‐tyrosine and L‐tryptophan residues as shown by an arrow in Figure 4. It has been noted that the binding of PG to serum albumin causes structural changes in albumin.

## 6 CONCLUSION

TBHQ, BHA, and PG possess high antioxidant and multifunctional characteristics and are broadly utilized in various fields such as food products, pharmaceuticals, and cosmetics. Therefore, the study of cytotoxicity and genotoxicity effects and their binding with biological molecules like serum albumin is highly demanded. TBHQ, BHA, and PG could be metabolized in the human gastric system and directly enter into biological routes through eating foods, drugs, and numerous products and may lead to alimentary deficiency and many illnesses. The toxicity effects of the above‐mentioned food antioxidants on human health are confirmed at amounts higher than the permissible limit. Previous studies have presented an effective combination of synthetic phenolic antioxidants with various purposes in food products. However, more studies on the use of these synthetic antioxidants in different industries are needed. In this context, comprehensive studies may be required, and they can be considered for shifting the outlook of industries toward the application of TBHQ, BHA, and PG as harmless antioxidants. The mentioned antioxidants have toxicity effects on human health via cyto/genotoxicity, interaction with albumin, and the initiation of inflammatory routes, metabolic disorders, augmented of diabetes. Recent studies result in this overview revealed that TBHQ, BHA, and PG antioxidants have cytotoxic, genotoxic, and carcinogenic effects if absorbed in high doses through the gastrointestinal tract. Thermodynamic parameters showed that the hydrophobic binding plays a major role in the complexation of the TBHQ, BHA, and PG with albumin. The molecular modeling results indicated that subdomain IIA plays a major role in the binding of TBHQ, BHA, and albumin. Finally, based on the aforementioned studies, we can suggest that TBHQ, BHA, and PG synthetic food antioxidants intake in human diets via the digestive system should be decreased.

## CONFLICT OF INTEREST STATEMENT

There are no conflicts of interest to declare.

## ETHICAL APPROVAL

This study does not involve any human or animal testing.

## Data Availability

No datasets were generated or analyzed during the current study.
